# Relationship Between Serum Levels of Oxidized Lipoproteins, Circulating Levels of Myeloperoxidase and Paraoxonase 1, and Diet in Young Subjects with Insulin Resistance

**DOI:** 10.3390/nu16223930

**Published:** 2024-11-18

**Authors:** Yaquelin Marchán-Figueroa, Brenda Tepec-Casarrubias, Ulises de la Cruz-Mosso, Constanza Cecilia Astudillo-López, Inés Matia-García, Lorenzo Salgado-Goytia, Mónica Espinoza-Rojo, Natividad Castro-Alarcón, Eugenia Flores-Alfaro, Isela Parra-Rojas

**Affiliations:** 1 Laboratorio de Investigación en Obesidad y Diabetes, Facultad de Ciencias Químico Biológicas, Universidad Autónoma de Guerrero, Chilpancingo de los Bravo 39087, Guerrero, Mexico; yaquelin.marfig@hotmail.com (Y.M.-F.); b_tepec@outlook.com (B.T.-C.); cony.astudillo.l@gmail.com (C.C.A.-L.); 15325@uagro.mx (I.M.-G.); 00636@uagro.mx (L.S.-G.); 2 Instituto de Neurociencias Traslacionales, Departamento de Neurociencias, Centro Universitario de Ciencias de la Salud, Universidad de Guadalajara, Guadalajara 44340, Jalisco, Mexico; ulises_cdm@hotmail.com; 3 Laboratorio de Biología Molecular y Genómica, Facultad de Ciencias Químico Biológicas, Universidad Autónoma de Guerrero, Chilpancingo de los Bravo 39087, Guerrero, Mexico; monicaespinoza@uagro.mx; 4 Laboratorio de Investigación en Microbiología, Facultad de Ciencias Químico Biológicas, Universidad Autónoma de Guerrero, Chilpancingo de los Bravo 39087, Guerrero, Mexico; natividadcastro@uagro.mx; 5 Laboratorio de Investigación en Epidemiología Clínica y Molecular, Facultad de Ciencias Químico Biológicas, Universidad Autónoma de Guerrero, Chilpancingo de los Bravo 39087, Guerrero, Mexico; eugeniaflores@uagro.mx

**Keywords:** ox-LDL, ox-HDL, diet, myeloperoxidase, paraoxonase 1, insulin resistance

## Abstract

Oxidized low-density lipoproteins (ox-LDLs) are involved in atherosclerotic plaque formation and progression and have been linked to insulin resistance (IR). Myeloperoxidase is a potent oxidant of lipoproteins related to atherogenic risk. High-density lipoproteins (HDLs) are considered antioxidants due to their association with paraoxonase 1 (PON1). However, HDL can also be oxidized (ox-HDL), and its relationship with IR has not been described. This study evaluated the relationship between circulating levels of myeloperoxidase and paraoxonase 1, diet, and serum levels of ox-LDL and ox-HDL in young people with IR. This cross-sectional study examined 136 young subjects (67 and 69 with and without insulin resistance, respectively). Serum levels of ox-LDL, ox-HDL, myeloperoxidase, and PON1 were quantified using an enzyme-linked immunosorbent assay. The nutritional dietary content of the foods was determined with a food frequency questionnaire, which was analyzed with Nutrimind 2013 software. Serum ox-HDL levels were higher in young subjects without IR than those with IR (*p* = 0.031). Women with IR presented increased ox-LDL levels compared with women without IR (*p* = 0.012) and men with IR (*p* < 0.001). In the IR group, serum ox-LDL levels were negatively correlated with total cholesterol, triglycerides, and LDL-C, whereas the correlation was positive in the insulin-sensitive group. Consumption of vitamins B1 and B2 was related to increased HDL-C levels, while higher ox-LDL levels were related to vitamin K intake. In addition, low energy consumption and phosphorus increased PON1 levels. The results suggest that insulin resistance in young women may promote lipoprotein oxidation, and the intake of B complex vitamins may have an antiatherogenic effect.

## 1. Introduction

Insulin resistance (IR) is one of the main causes of type 2 diabetes mellitus, which is one of the diseases with the highest prevalence in Mexico, with 16.9% in people aged 20 to 79 years [1]. IR is a metabolic alteration in which cells do not respond adequately to insulin stimulus [2], explaining its relation to hyperglycemia, dyslipidemia, hypertension, endothelial dysfunction, and a prothrombotic state [3]. Furthermore, IR contributes to the development of cardiovascular diseases, such as atherosclerosis, via several mechanisms, including increasing low-density lipoprotein (LDL) levels [4]. LDL particles, composed of lipids and proteins, mainly transport cholesterol to tissues. Under hypertriglyceridemic conditions, LDL particles become small and dense, making them more susceptible to oxidation and, thus, proatherogenic [5]. LDL oxidation occurs in the subendothelial tissue under oxidative stress conditions via oxidants generated by the enzymes lipoxygenase, nicotinamide adenine dinucleotide phosphate (NADPH) oxidase, and myeloperoxidase (MPO). The latter two, alongside endothelial nitric oxide synthase (eNOS), significantly contribute to LDL oxidation [6].

Myeloperoxidase is an important component of the innate immune system, secreted by neutrophils and monocytes [7]. MPO catalyzes the formation of hypochlorous acid (HOCl), a powerful oxidant that reacts with various types of proteins, lipids, and nucleic acids [8]. In chronic inflammatory processes, MPO and its oxidant products, such as HOCl, are associated with atherosclerosis [9]. This is because HOCl modifies the apolipoprotein B-100 of LDL and is related to the formation of hydroperoxides, chlorohydrins, and chlorinated fatty acids, among others, which leads to their recognition and accumulation in macrophages and the development of atherosclerotic plaque [10]. MPO levels are higher in subjects with IR than in overweight subjects without IR [11].

High-density lipoprotein cholesterol (HDL-C) possesses antioxidant, anti-inflammatory, and antithrombotic properties, making it antiatherogenic [9]. These properties are because it is associated with paraoxonase 1 (PON1), an enzyme that hydrolyzes lipid peroxides in oxidized LDL (ox-LDL) and breaks down phospholipid peroxidation products [12]. PON1 levels are reportedly lower in people with IR than those without IR [13].

Hypochlorous acid can also lead to oxidized HDL (ox-HDL) formation, decreasing HDL’s ability to mediate reverse cholesterol transport [14]. It also alters PON1’s structure, reducing its lactonase and antiperoxidation activities, thereby contributing to atherosclerosis progression [15].

The Western diet is characterized by high consumption of saturated fats, refined sugars, and proteins of animal origin, which is associated with increased oxidative stress, systemic inflammation, and LDL levels [16]. In addition, consuming sugars and fats is related to the development of IR via increased inflammation and oxidative stress [17]. Conversely, the Mediterranean diet, characterized by high consumption of fruits, vegetables, cereals, legumes, and nuts; moderate consumption of dairy products and fish; and low consumption of red meat, reduces oxidative stress levels by increasing antioxidant enzymes and, therefore, may prevent LDL oxidation [18].

Linna et al. (2015) reported higher ox-LDL levels in subjects with IR than those without IR [19]. However, ox-HDL levels have yet to be evaluated. Thus, this study aimed to analyze the relationship between serum levels of oxidized lipoproteins and diet, lipid profiles, and MPO and PON1 levels in young subjects with insulin resistance.

We hypothesized that young subjects with insulin resistance have high levels of oxidized LDL and HDL associated with a high-fat diet, high myeloperoxidase levels, and a low PON1 concentration.

## 2. Materials and Methods

### 2.1. Study Participants

A total of 136 young individuals from Guerrero, Mexico, were selected for this study, including 67 subjects with IR and 69 without IR. Among these participants, 80 were women, and 56 were men, with ages ranging from 18 to 30 years old. Blood pressure was measured by trained personnel. All participants answered a questionnaire to collect their personal information during an interview. Each individual provided information about lifestyle data, family history of diseases, physical activity, cigarette smoking, and medication use. The presence of diseases (liver, kidney, thyroid, cancer, and autoimmune diseases) was ruled out via a quick check-up. Current tobacco smoking was considered a daily intake of one or more cigarettes.

This study was approved by the Research Ethics Committee of the Autonomous University of Guerrero (CB-002/2018). All procedures were implemented per the ethical principles of the 2013 Declaration of Helsinki, and participants provided written informed consent before inclusion in this study.

### 2.2. Biochemical Parameters

Five-milliliter blood samples were drawn after 8–12 h of fasting. For serum samples, a 30 min period was allowed for clotting before serum separation, and they were centrifuged at 3000 RPM for 10 min. The serum samples were processed immediately. Levels of glucose, triglycerides, total cholesterol, and high-density lipoprotein cholesterol (HDL-C) were determined using conventional enzymatic assays with commercial kits (Spinreact, Santa Coloma, Spain) with an automated analyzer (Mindray, BS200, Hamburg, Germany). Low-density lipoprotein cholesterol (LDL-C) was measured using the direct method (Spinreact, Santa Coloma, Spain).

### 2.3. Insulin Serum Levels and IR Classification Criteria

Serum insulin concentrations were measured with an enzyme-linked immunosorbent assay using the Insulin Human ELISA kit (Invitrogen, Waltham, MA, USA, KAQ1251), following the manufacturer’s instructions. Absorbance was read at 450 nm using a plate reader (Multiskan FC, Thermo Fisher Scientific, 357, Shanghai, China). Insulin resistance was assessed using the homeostasis model assessment for insulin resistance (HOMA-IR) [glucose (mg/dL) × insulin (µU/mL)/405]. The subjects were categorized by IR status, with cutoff values of ≥3.2 and <3.2 for subjects with IR (the IR group) and without IR (the control group).

### 2.4. Ox-HDL, Ox-LDL, MPO, and PON 1 Levels

Serum levels of ox-HDL (MyBioSource, San Diego, CA, USA, MBS706079), ox-LDL (MyBioSource, USA, MBS265658), MPO (Abcam, Waltham, MA, USA, ab119605), and PON1 (Aviscera Bioscience, Santa Clara, CA, USA, SK00141-01) were measured with an enzyme-linked immunosorbent assay, according to the manufacturer’s instructions. The human oxidized low-density lipoprotein kit had a minimum detectable limit of up to 12 ng/mL, no cross-reaction with other factors, an intra-assay precision of <8%, and a detection range of 31.2–2000 ng/mL. The human oxidized high-density lipoprotein kit had high sensitivity (less than 7.81 ng/mL), no significant cross-reactivity or interference with other factors, an intra-assay precision of <8%, and a detection range of 31.25–2000 ng/mL. The human paraoxonase 1 kit had a sensitivity of 100 pg/mL, no cross-reaction with other factors, an intra-assay precision of 4–6%, and a standard range of 1.56–100 ng/mL.

### 2.5. Nutritional Status Assessment

Body composition assessment was performed using electrical bioimpedance, with light clothing and without shoes, using a body composition monitor (TANITA, MC-780U, Tokyo, Japan).

The average intake of energy, macronutrients, and micronutrients, such as vitamins and minerals, and the content of foods for each person were calculated using Nutrimind 2013 software based on the participants’ reported food consumption frequencies in questionnaires. For this purpose, the average daily intake of each food item, consumed in the six months preceding this study, was calculated in grams per day.

Nutritional assessments were based on the recommended macronutrient intake: 55–63% carbohydrates, <15% proteins, and <30% lipids [20]. Sugar consumption included sugar from food and added sugar. Nutrient consumption was classified as high or low according to the dietary reference intake (DRI) per the NOM-051-SCFI/SSA1-2010 based on the nutrient intake recommendations for the Mexican population [21] and guidelines from the National Academy of Medicine of Mexico [20], the Salvador Zubiran National Institute of Medical Sciences and Nutrition [22], and the WHO/Food and Agriculture Organization of the United Nations (FAO) [23].

### 2.6. Physical Activity

The level of physical activity was obtained using the short form of the International Physical Activity Questionnaire (IPAQ), which determines three classifications: sedentary, moderate physical activity, and intense physical activity [24].

### 2.7. Statistical Analysis

The results were analyzed using the statistical program STATA version 16.0. Ox-LDL and ox-HDL levels were logarithmically transformed for analysis. Means (X^−^) ± standard deviations (SDs) were calculated for symmetric quantitative variables, while medians and interquartile ranges (p25th and p75th percentiles) were used for non-symmetric variables. Group comparisons were performed using Student’s *t*-test, the Mann–Whitney U test, and the Kruskal–Wallis test. Qualitative variables were expressed as absolute and relative frequencies, with group differences assessed using the chi-square test or Fisher’s exact test. Correlation coefficients were determined via Spearman correlation, and associations were determined using multiple linear regression analysis. *p*-values of <0.05 were considered statistically significant.

## 3. Results

The median age of the 136 subjects selected was 20 years. Anthropometric characteristics; systolic blood pressure; levels of glucose, cholesterol, triglycerides, and LDL-C; and the LDL-C/HDL-C ratio were significantly higher (*p* < 0.001) in the insulin resistance (IR) group, while HDL-C levels were significantly lower (*p* < 0.001) compared with the control group (see Table 1).

**Figure 1:**
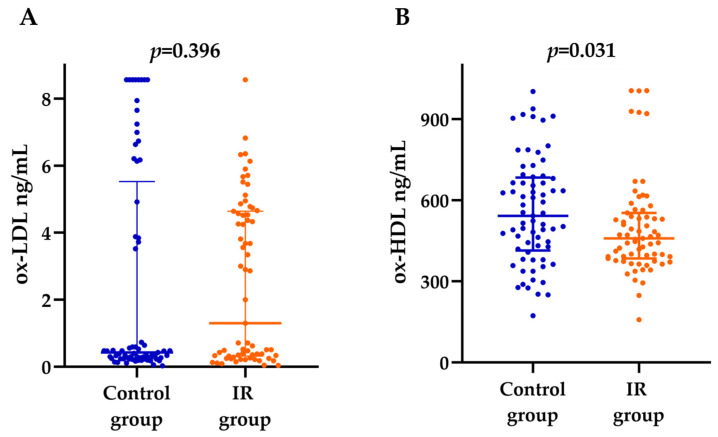
Serum levels of ox-LDL and ox-HDL in subjects with and without IR. (**A**) No significant difference was observed in ox-LDL levels between cases and controls. (**B**) Ox-HDL levels were higher in youths without IR. Data are presented as medians and 25th–75th percentiles. *p*-values were obtained using the Mann–Whitney U test.

No significant differences were observed in the median ox-LDL levels between youths with and without IR (1.3 (0.3–4.6) and 0.4 (0.3–4.9) ng/mL, respectively) (Figure 1A). Conversely, ox-HDL levels were significantly higher in youths without IR (542.6 (419.2–681) vs. 458.6 (385–553) ng/mL, respectively) compared with the control group (Figure 1B).

**Figure 2:**
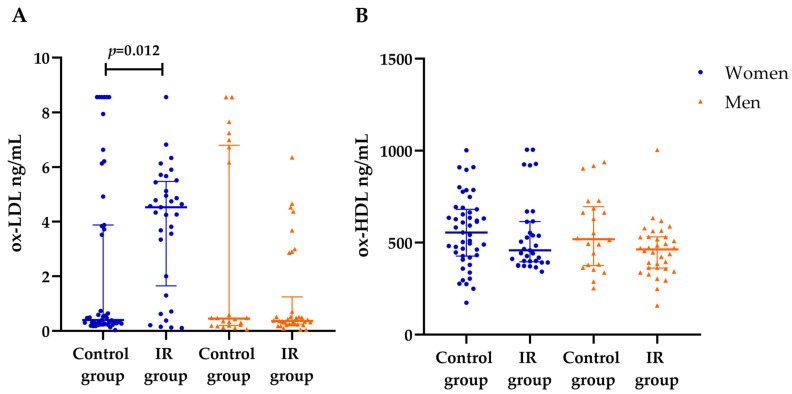
Serum levels of ox-LDL and ox-HDL in subjects with and without IR, separated by sex. (**A**) Ox-LDL levels were higher in women with IR compared with men. (**B**) There was no significant difference in ox-HDL levels between groups. Data are presented as medians and 25th–75th percentiles; *p*-values were calculated using the Mann–Whitney U test.

When compared by sex, Ox-LDL levels were found to be higher in women with IR (4.5 (2–5.4 ng/mL)) than in women without IR (0.4 (0.3–3.9 ng/mL)) (Figure 2A). Furthermore, ox-HDL levels showed no significant difference between the groups (Figure 2B).

Serum levels of MPO and PON1 were assessed, revealing no significant difference in the median MPO levels between subjects with and without IR (10.2 (7.2–20) ng/mL vs. 13.8 (9.4–17.3) ng/mL, respectively). Similarly, PON1 levels were comparable between the insulin-resistant group (272 (265.8–289.6) ng/mL) and the non-resistant group (270.1 (260.5–280.7) ng/mL). Likewise, no significant correlations were found between MPO and PON1 levels and ox-HDL or ox-LDL.

Analysis of the effects of lipid, MPO, and PON1 levels on oxidized lipoproteins in subjects with IR and the control group revealed that triglycerides were correlated with both types of oxidized lipoproteins in subjects with IR. Total cholesterol levels were associated with ox-LDL and ox-HDL in subjects with and without IR. Both HDL-C and LDL-C levels were associated with ox-LDL in the non-resistant group, whereas HDL-C was inversely associated with ox-HDL in subjects with IR (Table 2).

Upon evaluating the average daily intake of nutrients, it was observed that subjects with IR consumed significantly higher amounts of protein (Table 3).

The lipid profile, oxidized lipoprotein, and enzyme levels were compared with the type of nutrient consumption in young individuals. Total cholesterol levels were higher in those with an adequate intake of iron and potassium compared with those with a low intake. HDL-C levels were higher in subjects who had high consumption of vitamins B1 and B2 (Table 4). Subjects consuming a high percentage of protein had higher triglyceride levels, while LDL-C levels showed no significant differences between the groups.

Adequate intakes of carbohydrates and vitamin A, alongside low consumption of vitamin K, were related to an increase in ox-LDL levels. Conversely, low energy and phosphorus consumption was linked to increased PON1 levels (Table 4). No significant differences were observed in ox-HDL and MPO levels between the groups.

## 4. Discussion

Like previous studies [25,26], this research observed increased anthropometric measurements in youth with IR; increased cholesterol, triglyceride, and LDL-C levels; and decreased HDL-C levels. It has been shown that IR induces lipolysis in adipose tissue, leading to an increase in free fatty acids [4]; these promote enhanced VLDL and LDL production in the liver. In addition, it increases cholesterol transfer from HDL to apoB-containing lipoproteins, alongside an increase in hepatic lipase and lipoprotein lipase activity, resulting in decreased HDL-C levels [27].

Reactive oxygen species (ROS) at low concentrations play important roles in cell signaling. Their regulation is carried out by antioxidant molecules such as enzymes and vitamins. However, when ROS production exceeds antioxidant defense, oxidative stress occurs, which promotes lipid peroxidation and inflammation, important mechanisms in LDL oxidation [28,29]. In IR, compensatory hyperinsulinemia increases pro-oxidant enzyme levels, such as NADPH oxidase, which increases ROS production [30].

This research revealed that subjects without IR presented higher ox-HDL levels than subjects with IR. The role of ox-HDL in cardiovascular disease development has been debated, with Valiyaveettil et al. (2008) reporting that ox-HDL has a protective effect due to its antithrombotic activity. Zhang et al. (2022) found that ox-HDL favors the development of atherosclerosis because it promotes an increase in lipid uptake by macrophages [31,32]. The higher levels of ox-HDL in youth without IR can be attributed to its dependence on native HDL-C levels, which are absent in metabolic disease. Hence, youth with IR have lower levels of HDL-C; consequently, the oxidized form also has a low concentration [33].

ox-LDL levels were higher in women with than those without IR, a finding not previously reported. However, it has been described that estrogens can shorten the LDL oxidation time and increase antioxidant enzyme activity [34,35], while androgens increase lipid peroxidation [36]. In women, compensatory hyperinsulinemia decreases estrogen production and fosters hyperandrogenism; hence, this deregulation could increase ox-LDL levels in IR [37].

Association analyses demonstrated that cholesterol increases ox-HDL levels. Some studies indicate that high cholesterol levels are related to increases in reactive oxygen species and lipid peroxidation [38,39]. Furthermore, HDL-C levels presented a negative association with ox-HDL levels. This was because HDL has an antioxidant function mediated by various apolipoproteins, lipid transfer proteins, and associated enzymes [40]. Apo A-I is the main apolipoprotein that comprises HDL. One of its important functions is preventing the accumulation of lipid peroxides in LDL and HDL [41].

MPO levels showed no association with ox-LDL and ox-HDL levels, attributed to lipoprotein oxidation being influenced not only by MPO but also by reactive oxygen species, metal ions, lipoxygenases, and other free radicals [42]. Conversely, no relationship was found between PON1 levels, ox-LDL, and ox-HDL. Therefore, MPO and PON1 levels are only indicative of the presence of the enzymes, and their activity in youth with IR should be evaluated to determine the relationship with oxidized lipoproteins in this population.

In this study, it was found that serum PON1 levels were not related to exercise habits. This could be because PON1 levels increase immediately after exercise and after 24 h, return to their basal levels [43]; however, more studies are needed to investigate the effect of exercise on PON1 concentrations, since most research so far focuses on measuring activity. On the other hand, we also found no relationship of PON1 concentration with current tobacco smoking; this is probably because in this study, in the group of non-smokers, ex-smokers could have been included and have been shown to have lower PON1 levels (similar to current smokers) than subjects who have never smoked [44].

This research also showed that subjects with IR had a higher intake of both animal- and plant-derived proteins. Similar studies have reported that high protein consumption increases the risk of diabetes; however, whether the detrimental effects of protein on IR and diabetes are due to its consumption or the nutrients accompanying it remains unknown [45].

Intake of vitamins B1 and B2 was associated with increased HDL-C levels. Supplementation with B vitamins in coronary artery disease patients has been shown to increase HDL-C levels by up to 16.7%, along with reductions in total cholesterol and triglyceride levels [46].

Higher triglyceride levels were observed in young subjects with high protein consumption, suggesting an effect from animal proteins. Red meat consumption is related to increased triglyceride levels [47].

Furthermore, carbohydrate consumption was related to increased ox-LDL levels. Välimäki et al. (2012) demonstrated that carbohydrate consumption can increase ox-LDL levels by 28–33% [48]. A possible mechanism is that carbohydrates promote the synthesis of triglycerides, which contributes to the increase in small and dense subclasses of LDL that are more susceptible to oxidation [49]. Likewise, low vitamin K consumption was related to increased serum ox-LDL levels. Research has found that vitamin K has antioxidant activity due to its ability to inhibit the activation of lipoxygenase and prevent the accumulation of free radicals. It also presents anti-inflammatory activity, which means low consumption of this vitamin may be related to increased oxidative stress and inflammation and, consequently, increased LDL oxidation [50].

Finally, higher serum PON1 levels were found in young subjects with lower energy consumption, aligning with evidence that foods like olive oil, fruits, and vegetables boost serum PON1 levels, which are the main components of the Mediterranean diet [51]. This is consistent with the lower consumption of fruits and vegetables reported by study participants.

This research underscored the significant influence of sex on atherogenic risk via elevated ox-LDL levels in IR and identified dietary nutrients impacting lipid profiles and serum lipoprotein levels. However, a study limitation was the lack of consideration for food preparation methods, which can affect serum oxidized lipoprotein levels, especially with the use of repeatedly heated oils known to generate oxidative stress and inflammation [52]; therefore, subsequent studies should consider the use of these oils. The strength of this study is related to its rigorous evaluation of lifestyle factors, including physical activity and nutritional status. Finally, future research should quantify the serum levels of vitamins related to ox-LDL levels to corroborate the effects observed in this study and, subsequently, search for the mechanisms that lead to this relationship.

## 5. Conclusions

Insulin resistance in women is related to increased ox-LDL levels, a relationship not observed in men. Additionally, the intake of B vitamins favors an increase in HDL-C levels, while low energy and phosphorus consumption increases PON1 levels. Therefore, it is important for young subjects to adopt a healthy diet, and supplementation with B vitamins may be suggested to prevent atherogenic dyslipidemia.

## Figures and Tables

**Table 1:** Anthropometric and biochemical characteristics of young subjects with and without insulin resistance.

Characteristics	Total (*n* = 136)	Control Group (*n* = 69)	IR Group (*n* = 67)	*p*-Value
Sex *n* (%)				
Women	80 (58.8)	47 (68.1)	33 (49.2)	0.025 ^a^
Men	56 (41.2)	22 (31.9)	34 (50.7)	
Age (years)	20.5 (19–22)	20 (19–21)	21 (19–22)	0.089 ^b^
Physical activity				
Sedentary	86 (63.3)	50 (72.5)	36 (53.7)	
Moderate activity	32 (23.5)	13 (18.8)	19 (28.4)	0.068 ^a^
Intense activity	18 (13.2)	6 (8.7)	12 (17.9)	
Smoking status				
No	114 (83.8)	61 (88.4)	53 (79.1)	0.141 ^a^
Yes	22 (16.2)	8 (11.6)	14 (20.9)	
Weight (kg)	59.9 (52.7–78)	55.8 (49.6–60)	76.7 (59.8–92.6)	<0.001 ^b^
Height (cm)	162.2 ± 9	160.6 ± 8.4	163.8 ± 9.3	0.038 ^c^
BMI (kg/m^2^)	23.8 (20.9–30)	21.6 (19.7–23.7)	29.6 (24–33.1)	<0.001 ^b^
Waist circumference (cm)	82 (74.3–95.6)	76 (72–81.5)	95.3 (83–106)	<0.001 ^b^
Hip circumference (cm)	98 (91.5–108.9)	93 (90–99)	108 (98–113)	<0.001 ^b^
Fat mass (kg)	16.1 (11.9–25)	12.8 (10.2–16.1)	24.6 (16.5–31)	<0.001 ^b^
Fat percentage	27.1 ± 7.7	23.4 ± 6.6	30.9 ± 6.9	<0.001 ^c^
Waist-to-hip ratio	0.85 (0.8–0.9)	0.8 (0.8–0.9)	0.9 (0.8–0.9)	<0.001 ^b^
SBP (mmHg)	110.9 ± 13	107.3 ± 10.7	114.6 ± 14.2	<0.001 ^c^
DBP (mmHg)	68.4 ± 9.8	66.8 ± 8.9	70.1 ± 10.5	0.057 ^c^
Glucose (mg/dL)	84 (77–90.5)	78 (72–84)	89 (84–95)	<0.001 ^b^
Triglycerides (mg/dL)	91 (66–151)	69 (56–89)	148 (96–194)	<0.001 ^b^
Total cholesterol (mg/dL)	151.5 (131–180)	138 (128–164)	171 (142–202)	<0.001 ^b^
HDL-C (mg/dL)	39 (36–49)	44 (38–55)	38 (33–43)	<0.001 ^b^
LDL-C (mg/dL)	95.5 (78–118)	87 (71–98)	113 (85–142)	<0.001 ^b^
LDL-C/HDL-C ratio	2.3 (1.7–3.1)	1.8 (1.5–2.5)	2.9 (2–3.4)	<0.001 ^b^
Insulin (µU/mL)	16 (9–25.8)	9.3 (5.5–11.9)	26.1 (19.5–32.3)	<0.001 ^b^

Data are presented as absolute frequencies (%), medians (p25th–p75th), and means ± standard deviations (SDs). *p*-values were derived using ^a^ chi-square test, ^b^ Mann–Whitney U test, and ^c^ Student’s *t*-test. *p*-values of <0.05 were considered statistically significant. Abbreviations: IR, insulin resistance; BMI, body mass index; SBP, systolic blood pressure; DBP, diastolic blood pressure; HDL-C, high-density lipoprotein cholesterol; LDL-C, low-density lipoprotein cholesterol.

**Table 2:** Association of lipid profiles and MPO and PON1 levels with oxidized lipoproteins in subjects with and without IR.

	Control Group				IR Group			
	Ox-LDL		Ox-HDL		Ox-LDL		Ox-HDL	
	β (95% CI)	*p*-Value	β (95% CI)	*p*-Value	β (95% CI)	*p*-Value	β (95% CI)	*p*-Value
Triglycerides (mg/dL)	0.02 (−0.01, 0.04)	0.153	0.49 (−1.03, 2.01)	0.524	−0.02 (−0.03, −0.01)	<0.001	−0.84 (−1.64, −0.04)	0.040
Total cholesterol (mg/dL)	0.05 (0.02, 0.08)	<0.001	2.06 (0.42, 3.71)	0.015	−0.02 (−0.03, −0.01)	0.003	−1.04 (−2.01, −0.06)	0.038
HDL-C (mg/dL)	−0.13 (−0.21, −0.06)	0.001	−1.12 (−6.30, 4.06)	0.667	−0.01 (−0.07, 0.06)	0.859	−7.66 (−13.39, −1.93)	0.010
LDL-C (mg/dL)	0.06 (0.02, 0.09)	0.002	1.60 (−0.70, 3.89)	0.169	−0.01 (−0.02, 0.01)	0.300	−0.55 (−1.60, 0.50)	0.299
MPO (ng/mL)	0.07 (−0.8, 0.22)	0.341	−2.16 (−11.13, 6.81)	0.632	0.02 (−0.24, 0.29)	0.844	−3.45 (−35.95, 29.06)	0.804
PON1 (ng/mL)	−0.01 (−0.03, 0.002)	0.089	0.33 (−0.67, 1.33)	0.518	0.01 (−0.03, 0.05)	0.565	2.01 (−2.51, 6.52)	0.318

Linear models were adjusted for sex, age, and BMI. Data are presented as β-coefficients and 95% confidence intervals. *p*-values of <0.05 were considered significant. Abbreviations: IR, insulin resistance; ox-LDL, oxidized low-density lipoprotein; ox-HDL, oxidized high-density lipoprotein; HDL-C, high-density lipoprotein cholesterol; LDL-C, cholesterol low-density lipoprotein; MPO, myeloperoxidase; PON1, paraoxonase 1.

**Table 3:** Energy and nutrient intake in the studied groups.

Nutrient	DRI (U/day)	Total (*n* = 136)	Control Group (*n* = 69)	IR Group (*n* = 67)	*p*-Value
Energy (cal)	1500–2000	2677 (2138–3950)	2693.5 (2304–4101.5)	2665 (1878–3887)	0.477
Carbohydrates (%)	55–63	56 (52–60)	55.5 (53–61)	56 (49–60)	0.430
Proteins (%)	≤15	13 (11–15)	12 (11–14)	14 (12–15)	0.017
Lipids (%)	≤30	31 (27–35)	31 (27.5–34.5)	31 (27–36)	0.840
Cholesterol (mg)	<250	229.5 (171–297)	221.5 (181.5–284.5)	234 (168–326)	0.555
SFAs (g)	<22	21.5 (14–31)	20 (14.5–32)	22 (13–30)	0.949
MUFAs (g)	<34	25.5 (16–36)	26.5 (19.5–36.5)	25 (15–36)	0.770
PUFAs (g)	>22	10.5 (6–21)	12 (6–25.5)	8.5 (5–16)	0.116
Fiber (g)	≥30	30.5 (23–49)	33 (24–48.5)	28.5 (19–52)	0.216
Sugars (g)	26–50	63.5 (44–101)	65.5 (43.5–104.5)	61.5 (45–101)	0.979

Data are presented as medians (p25th–p75th percentiles); *p*-values were obtained using the Mann–Whitney U test. *p*-values of <0.05 were statistically significant. Abbreviations: DRI, dietary reference intake; IR, insulin resistance; SFAs, saturated fatty acids; MUFAs, monounsaturated fatty acids; PUFAs, polyunsaturated fatty acids.

**Table 4:** Comparison between dietary reference intake and HDL-C, ox-LDL, and PON1 levels in all participants.

Nutrients	HDL-C	*p*-Value	Ox-LDL	*p*-Value	PON1	*p*-Value
Energy (Cal)		0.299 ^a^		0.621 ^a^		0.008 ^a^
Low (<1500 cal)	36.5 (31–42)		2 (0.3–4.3)		289.9 (271–300.3)	
Adequate (1500–2000 cal)	40.5 (35.5–53)		4.26 (0.4–6)		268.5 (261.8–279.6)	
High (>2000 cal)	39 (35–48)		0.5 (0.3–5)		264.9 (255–273.5)	
**Macronutrients**						
Carbohydrates (%)		0.586 ^a^		0.026 ^a^		0.644 ^a^
Low (<55%)	38 (35–48)		0.6 (0.3–4.6)		266.7 (261.8–275)	
Adequate (55–63%)	39 (35–45)		0.7 (0.3–6.2)		268.9 (257.5–279.6)	
High (>63%)	39(37–60)		0.2 (0.1–0.4)		249.2(165.9–287.8)	
Proteins (%)		0.212 ^b^		0.662 ^b^		0.369 ^b^
Adequate (≤15%)	39 (36–48)		0.5 (0.3–5.5)		268.1 (257.6–274.7)	
High (>15%)	37 (34–43)		0.7 (0.3–5.1)		268.5 (261.8–293.2)	
Lipids (%)		0.936 ^b^		0.256 ^b^		0.359 ^b^
Adequate (≤30%)	39 (36–44)		0.5 (0.3–4.5)		265.2 (253.6–273.9)	
High (>30%)	38 (35–48)		2.9 (0.3–5.4)		270.2 (261.8–275.5)	
**Vitamins**						
Vitamin A (mcg)		0.303 ^b^		0.014 ^b^		0.489 ^b^
Low (M: <730; W: <570 mcg)	38 (35–46)		0.5 (0.2–4.5)		268.5 (257.4–275.1)	
Adequate (M: ≥730; W: ≥570 mcg)	42 (34–55)		3.7 (0.5–6.2)		267.7 (260.5–281.1)	
Vitamin B1 (mg)		0.020 ^a^		0.326 ^a^		0.298 ^a^
Low (<1 mg)	36 (33–38)		3.5 (0.4–5.9)		271 (268.5–289.9)	
Adequate (1 mg)	38 (34–48)		0.5 (0.2–4.5)		268.1 (258.5–275.1)	
High (>1 mg)	41 (37–52)		0.7 (0.4–6.1)		264.4 (254.3–275.2)	
Vitamin B2 (mg)		0.040 ^a^		0.264 ^a^		0.869 ^a^
Low (<1 mg)	32 (30.5–34)		0.5 (0.4–3.2)		264.1 (257.3–271)	
Adequate (1 mg)	38 (36–47)		0.4 (0.2–4.6)		268.5 (257.8–279.9)	
High (>1 mg)	39 (35–48)		1.3 (0.4–5.7)		267.7 (260.5–275.4)	
Vitamin K (mcg)		0.919 ^b^		0.007 ^b^		0.403 ^b^
Low (M: <100; W: <75 mcg)	39 (35–48)		3.2 (0.3–5.7)		268.5 (258.6–279.9)	
Adequate (M: ≥100; W: ≥75 mcg)	38 (37–46.5)		0.3 (0.2–0.5)		264.4 (261.4–271.7)	
**Minerals**						
Calcium (mg)		0.798 ^b^		0.691 ^b^		0.343 ^b^
Low (<1000 mg)	38 (35–48)		0.5 (0.3–4.6)		268.5 (261.8–281.1)	
Adequate (≥1000 mg)	39 (35–45)		0.6 (0.3–5.7)		265.9 (254.3–274.9)	
Phosphorus (mg)		0.187 ^b^		0.070 ^b^		0.003 ^b^
Low (<700 mg)	37 (33–46)		4.3 (0.5–6.3)		279.9 (270.5–300.3)	
Adequate (≥700 mg)	39 (35–49)		0.5 (0.3–4.9)		265.1 (257.4–273.7)	
Iron (mg)		0.685 ^b^		0.888 ^b^		0.281 ^b^
Low (M: <15; W: <21 mg)	38 (35.5–46.5)		1.8 (0.3–5.2)		270.2 (258.5–284.5)	
Adequate (M: ≥15; W: ≥21 mg)	39 (35–48)		0.5 (0.3–5.5)		264.9 (258.6–273.5)	
Magnesium (mg)		0.133 ^b^		0.138 ^b^		0.398 ^b^
Low (M: <320; W: <250 mg)	37 (34.5–46)		3.5 (0.4–5.8)		270.4 (259.3–279.9)	
Adequate (M: ≥320; W: ≥250 mg)	39 (36–52)		0.5 (0.2–4.7)		265.2 (258.6–273.9)	

Data are presented as medians (p25th–p75th percentiles); *p*-values were obtained using ^a^ Kruskal–Wallis and ^b^ Mann–Whitney U tests. Abbreviations: M, dietary reference intake for men; W, dietary reference intake for women; HDL-C, high-density lipoprotein cholesterol; ox-LDL, oxidized low-density lipoprotein; PON1, paraoxonase 1.

## Data Availability

The data used to support the findings of this study are available from the corresponding author upon reasonable request.
